# Molecular Mechanisms Underlying CRISPR/Cas-Based Assays for Nucleic Acid Detection

**DOI:** 10.3390/cimb45010043

**Published:** 2023-01-10

**Authors:** Denis N. Antropov, Grigory A. Stepanov

**Affiliations:** Institute of Chemical Biology and Fundamental Medicine, Siberian Branch of the Russian Academy of Sciences, 630090 Novosibirsk, Russia

**Keywords:** genome editing proteins, isothermal amplification, nucleic acid detection systems, diagnostics, Cas proteins

## Abstract

Applied to investigate specific sequences, nucleic acid detection assays can help identify novel bacterial and viral infections. Most up-to-date systems combine isothermal amplification with Cas-mediated detection. They surpass standard PCR methods in detection time and sensitivity, which is crucial for rapid diagnostics. The first part of this review covers the variety of isothermal amplification methods and describes their reaction mechanisms. Isothermal amplification enables fast multiplication of a target nucleic acid sequence without expensive laboratory equipment. However, researchers aim for more reliable results, which cannot be achieved solely by amplification because it is also a source of non-specific products. This motivated the development of Cas-based assays that use Cas9, Cas12, or Cas13 proteins to detect nucleic acids and their fragments in biological specimens with high specificity. Isothermal amplification yields a high enough concentration of target nucleic acids for the specific signal to be detected via Cas protein activity. The second part of the review discusses combinations of different Cas-mediated reactions and isothermal amplification methods and presents signal detection techniques adopted in each assay. Understanding the features of Cas-based assays could inform the choice of an optimal protocol to detect different nucleic acids.

## 1. Introduction

Pathogen detection methods usually target viral or bacterial proteins or nucleic acids. A nucleic acid detection system is a multistep assay that includes (1) extraction of nucleic acids from a biospecimen; (2) isothermal amplification of a nucleic acid fragment to reach the detection threshold; (3) detection of nucleic acids and experimental data analysis. Classic polymerase chain reaction (PCR) [1,2] and real-time PCR [3] currently give way to advanced systems. These systems combine highly specific Cas-based protein complexes with an isothermal amplification step that allows for nucleic acid analysis under constant temperature. Originally, the clustered regularly interspaced short palindromic repeats (CRISPR)/Cas-system is an immune gene cluster that protects bacteria from invading pathogens [4]. Via gene recombination, the protective mechanism inserts a foreign nucleic acid sequence in the gene cluster, downstream to “direct repeats” that are recognized by a Cas protein. During translation, Cas proteins form a complex with their guide RNAs (gRNA) that are complementary to the specific fragments of nucleic acids. If designed artificially, modern CRISPR/Cas systems can incorporate diverse Cas proteins and specific gRNAs. Cas proteins specifically detect various nucleic acids (ss/ds DNA or RNA) and, thus, facilitate in vivo diagnostics. Another advantage of Cas proteins is their tolerance to contaminants, which supports reactions without preliminary purification of nucleic acids and reduces diagnostic time. A wide range of nucleic acid detection platforms varying in Cas protein types and methods of isothermal amplification has been developed. The most prominent systems are Cas9-based, such as CRISPR/Cas9-triggered isothermal exponential amplification reaction (CAS-EXPAR) [5], NASBA–CRISPR cleavage (NASBACC) [6], CRISPR–Cas9-triggered nicking endonuclease-mediated strand displacement amplification method (CRISDA) [7], Cas9-mediated lateral flow nucleic acids assay (CASLFA) [8], or RCA-assisted CRISPR/Cas9 cleavage (RACE) [9]. Cas12 and Cas14 are used in DNA endonuclease-targeted CRISPR trans reporter (DETECTR) [10], one-hour low-cost multipurpose highly efficient system (HOLMES) [11], and HOLMES v.2 [12]), while Cas13 is employed in specific high-sensitivity enzymatic reporter unLOCKing (SHERLOCK) [13], SHERLOCK v.2 [14], combinatorial arrayed reactions for multiplexed evaluation of nucleic acids (CARMEN) [15], SHERLOCK-based profiling of in vitro transcription (SPRINT) [16], and SHERLOCK and HUDSON integration to navigate epidemics (SHINE) [17]. This review covers molecular mechanisms underlying various CRISPR/Cas-based nucleic acid detection systems.

## 2. Sample Preparation Methods for the Downstream Nucleic Acid Detection

**Figure 1:**
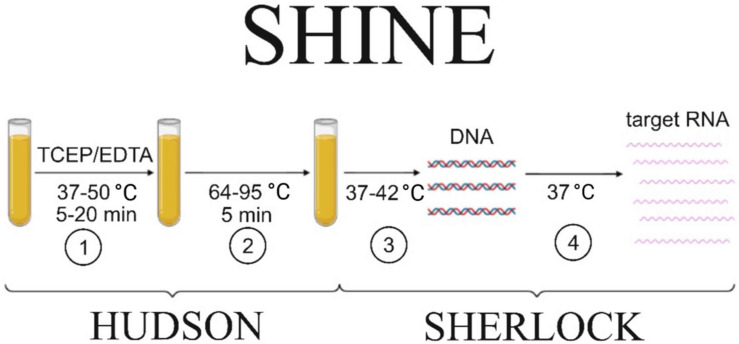
Scheme of viral particle detection in biological fluids using the SHINE protocol. The main steps are (1) nuclease inactivation to prevent nucleic acid degradation; (2) viral particle inactivation, facilitating the nucleic acid release from the particle; (3) RT–RPA step to amplify a sufficient amount of DNA; (4) target RNA production for the specific Cas13a-mediated detection. The steps that are included either in HUDSON or SHERLOCK protocols are indicated by the curly braces.

Nucleic acid detection in clinical specimens requires the inactivation of Rnases-specific RNA-cleaving enzymes that are abundant in biofluids. The heating unextracted diagnostic samples to obliterate nucleases (HUDSON) method [18] implies chemical inactivation of RNase by heating clinical specimens to 95 °C. In combination with SHERLOCK (SHINE), this approach allows for detecting up to 5–10 viral RNA copies in less than an hour (Figure 1) [17].

## 3. Methods of Isothermal Amplification

The next step of nucleic acid detection requires amplification of the target sequence. Currently, isothermal methods are being developed and used for rapid and field-deployable amplification.

**Figure 2:**
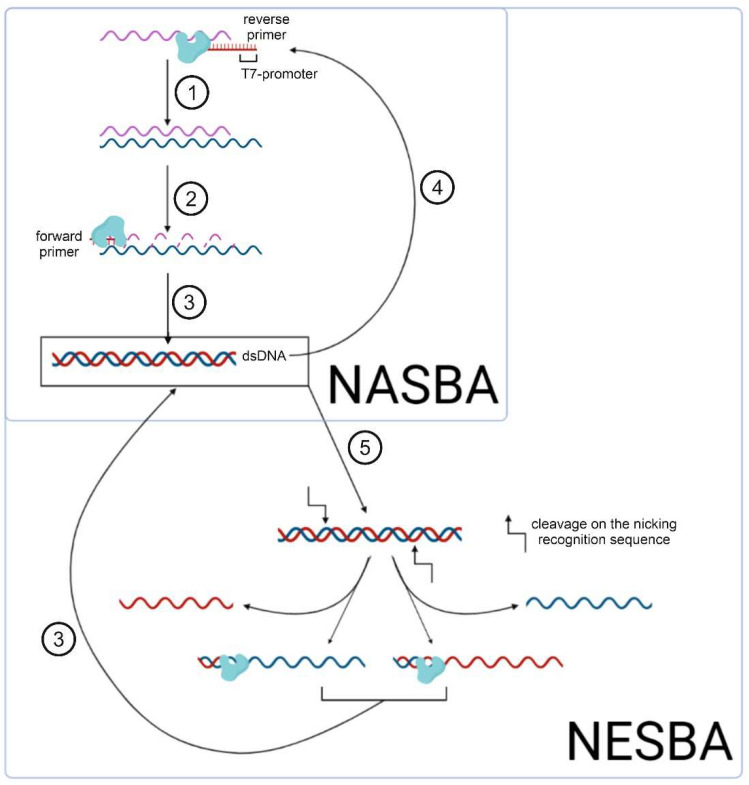
Scheme of isothermal amplification NASBA and its modification NESBA, including the following steps: (1) reverse transcription of the initial RNA fragment by the AMV reverse transcriptase (shown in the figure); (2) RNase H-mediated cleavage of these RNAs; (3) synthesis of dsDNA as a matrix for (4) subsequent in vitro transcription; (5) nicking of the dsDNA with its subsequent amplification.

Nucleic acid sequence-based amplification (NASBA) [19], recombinase polymerase amplification (RPA) [13], RT–RPA (the same as previous but combined with reverse transcription) [13], and loop-mediated isothermal amplification (LAMP) [20] are the most well-known and widely used methods among modern approaches to isothermal amplification. Standard NASBA (Figure 2) is a multi-enzyme reaction involving AMV reverse transcriptase, T7 RNA polymerase, and RNase H [19]. The method achieves 10^8^–10^9^-fold amplification of a target 100–200 nucleotide RNA product in 120 min reaction time [19]. The NASBA isothermal amplification reaction detects both bacterial [21,22,23,24] and viral [25,26,27,28] pathogens. Wu et al. [29] analyzed reaction conditions, such as primer concentration, reaction temperature, and reaction time. Their optimized protocol informed a new isothermal NASBA sequencing-based high-throughput test (INSIGHT method), which provides multiplexed pathogen detection through massively parallel next-generation sequencing. The NASBA reaction is, however, limited in its specificity, which is typical for multi-enzyme reactions. Hence, it is necessary to design specific probes and primers for the target amplicons [30,31,32], as well as to include a preliminary step for annealing at 65 °C.

In contrast, nicking and extension chain reaction system-based amplification (NESBA) refers to a more sensitive variation of NASBA (Figure 2). Ju et al. demonstrated that NESBA detects RNA fragments, encoding E and N genes of SARS-CoV-2, at a concentration as low as 0.5 copies/ul [33]. Here, the forward T7 primer is extended to form multiple copies of the DNA sequence that enter the T7 RNA polymerase-catalyzed transcription. Thus, compared to the original NASBA method, the NESBA reaction yields a significantly amplified signal, while achieving excellent sensitivity (100%) and specificity (100%).

**Figure 3:**
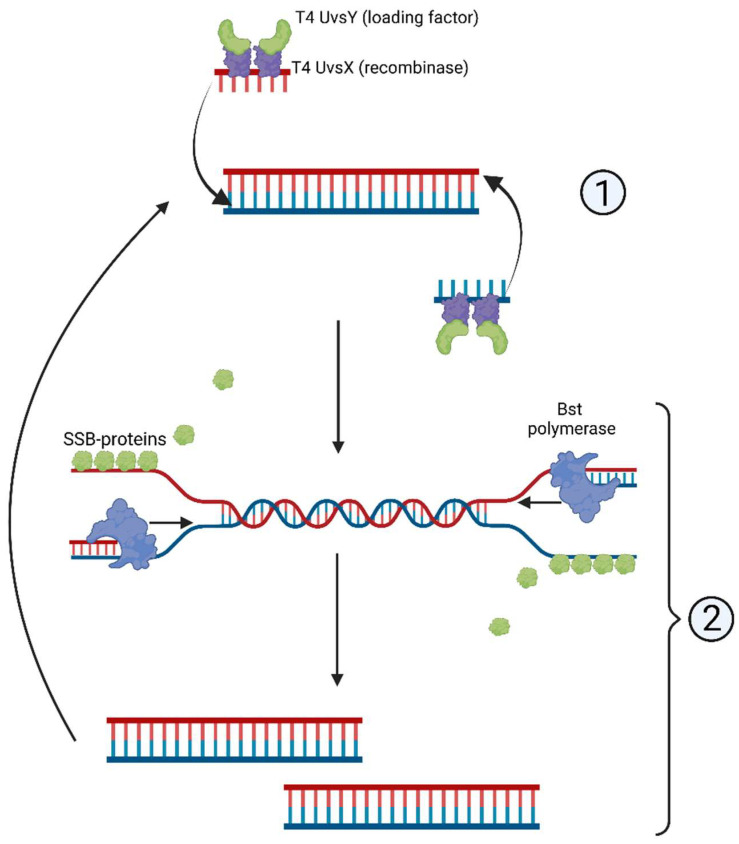
Scheme of recombinase polymerase amplification, RPA: (1) T4 UvsY- and T4 UvsX-mediated complementary strand recognition and primer annealing; (2) D-loop formation and synthesis of the complementary strand.

The RPA reaction, including its combination with Cas proteins, is used extensively for clinical diagnostics in a laboratory setting [34]. The RPA mechanism exploits the activity of homologous recombination proteins. T4 UvsX protein (recombinase) and T4 UvsY (loading factor) bind to the primers, enabling the search for homologous sequences in the template DNA [34]. The complex of primers and proteins creates a D-loop structure in double-stranded DNA, and unwinding DNA strands are stabilized by the single-strand binding (SSB) proteins T4gp32 [34] (Figure 3).

A distinct advantage of RPA lies in entirely isothermal amplification: it proceeds without any preliminary steps that would require elevated temperatures. RPA primers are typically longer than those designed for other isothermal methods [34]. Shorter primers, although used in some works, reportedly decrease RPA reaction rate and sensitivity [35,36]. Inhibitors such as hemoglobin (20 g/L), heparin (0.5 U), and urea (1.25%) are shown to not interfere with the reaction significantly [35,36]. Moreover, higher concentrations of hemoglobin (50 g/L), ethanol (4% *v*/*v*), or urea (up to 5%) reduce the reaction rate only slightly [37,38,39]. These data favor RPA over detergent-sensitive methods such as NASBA.

To detect a trace quantity of RNA in clinical specimens, RPA is coupled with Cas13-based interference (SHERLOCK, SHERLOCK v.2, CARMEN). Similarly, RT–RPA and LAMP are combined with Cas12-based DNA interference, constituting the SHERLOCK and SHERLOCK v.2 or DETECTR assays, respectively. LAMP is widely used in clinical diagnostics alongside RPA. This type of isothermal amplification is based on the synthesis of concatemeric amplicons from a 100–250 nucleotide DNA sequence, which is facilitated by the Bst DNA polymerase derived from *Bacillus subtilis*. Two or three pairs of primers are used specifically to amplify the target product [16,36]. To visualize the accumulation of the LAMP product in the visible light range (‘naked-eye visualization’), SYBR Green I is introduced to the reaction mix as a fluorescent dye with a high affinity for double-stranded DNA [40,41]. Drawbacks of LAMP include a high reaction temperature (60–65 °C), complex primer design, and a high probability of producing non-specific amplicons. Additionally, some non-specific products are amplified in the absence of the template because of the interaction between different primer pairs [42]. Many detection methods are currently available that utilize LAMP in a combination with Cas12 and Cas13 proteins that demonstrate specific activity. Additionally, a system named Cas14–DETECTR, which combines LAMP with Cas14 interference, was developed in 2018 [43].

Some detection assays rely on other approaches to isothermal amplification, such as strand displacement amplification (SDA) [9] or rolling circle amplification (RCA) [10]. In SDA, DNA polymerase that possesses strand displacement activity amplifies the DNA template. RCA enables the synthesis of multiple tandem copies from a single DNA strand, resulting in the amplification of a specific concatemeric product. The cross-priming amplification method (CPA) is similarly based on producing a concatemeric amplicon. It allows for amplifying a target sequence from four copies/μL of genomic DNA of *Mycobacterium tuberculosis* with neither preliminary heating nor nickase in the reaction [44]. However, a clear shortcoming of using the CPA test in the field is that it requires maintaining a high temperature (63–68 °C) to amplify a specific sequence. No reaction product is detected at temperatures below 58 °C [44].

To obtain a specific target product using the above-mentioned isothermal amplification methods, several (typically three) pairs of primers must be used, which considerably complicates the design of an assay. This necessitated the development of other isothermal amplification systems that would require only a pair of primers or just a single primer to achieve the same result in a comparable or even shorter amount of time. For example, polymerase spiral reaction (PSR) utilizing Bst polymerase has already been proven as a reliable detection method to visualize the reaction product by chemiluminescence [45]. Ji et al. applied this method to successfully detect DNA of the porcine circovirus (PSV-3) in clinical specimens. The detection limit reached about 100 copies/μL and there was no cross-reactivity with other circoviruses [45], indicating the high sensitivity and specificity of the method. PSR was found compatible with naked-eye detection based on a mixture of phenol red and cresol red dyes. The color of the mixture changes from pink to yellow if specimens contain the target DNA [45]. In another work [46], SYBR Green I is chosen for visualization in the visible range. PSR reaction mechanism leverages longer primers to obtain a product that contains complementary sequences and, hence, closes into a helix that is subsequently extended [47].

**Figure 4:**
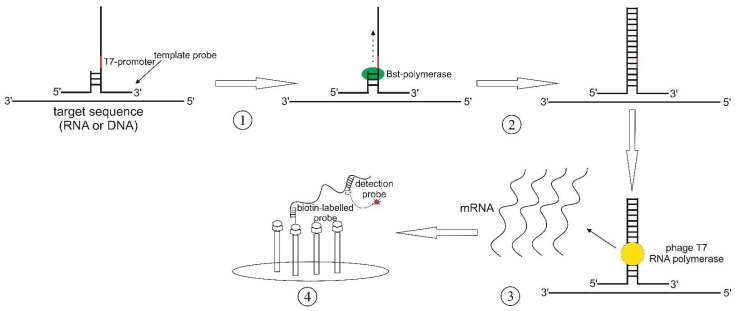
SMART scheme: (1) 3-way junction (3WJ) formation; (2) synthesis of the second strand by the Bst DNA polymerase; (3) synthesis of the specific mRNA; (4) detection of the synthesized RNA fragment by a specific detection probe that is annealed to a biotin-labeled probe.

The signal-mediated amplification of RNA technology (SMART) method employs a pair of “probes”, which are complementary to both each other and a specific sequence within the DNA or RNA template (Figure 4). Here, a T-like three-way junction (3WJ) structure is formed. It is then recognized by the Bst polymerase, which extends the ssDNA fragment to form dsDNA that incorporates a promotor of the T7-phage RNA polymerase [48]. Hall et al. were able to specifically detect the mRNA of cyanophages, targeting cyanobacteria *Synechococcus* sp., even 10 h after transfection [49]. A specific signal was also obtained for similar genes in cyanophages. SMART required neither extraction nor purification of genomic DNA since the detection was performed in cell lysates. The data obtained suggest the high specificity of the SMART system as well as its tolerance to the detergents present in the reaction mix.

Unusual (UIMA) and linear-target (LIMA) isothermal multimerization and amplification systems exploit strand-displacing polymerases that possess reverse transcriptase activity but lack 5′–3′-exonuclease activity. An example of such a polymerase is the Bst polymerase used in LAMP isothermal amplification. Due to its activity, the reaction products comprise long sequences of a multiplied repeated target DNA fragment. Yet, these reactions require a single pair of primers or, as in the case of UIMA, only one primer. The specificity of one-primer reactions has been proven in experiments where the reaction mix contained either no FAM-labeled template or no FAM-labeled primer. No product amplification has been observed on the real-time fluorescence-tracked electropherogram, indicating the absence of contamination [50]. A single primer is sufficient owing to unique reaction conditions. Under high concentrations of Mg^2+^ (8–10 mM), the primer–template complex tends to form loop structures [50], in which the mismatches are extended by the aforementioned polymerases to form dsDNA. During amplification, the product is extended by one segment, and complementary sequences from different fragments are further extended at the annealing step. Isothermal amplification enables a 10^6^–10^10^-fold increase in the concentration of the target nucleic acid fragment in clinical specimens over a relatively short time (5 min to 1 h) and at a constant temperature (25–65 °C). Hence, it allows a sample containing as few as 1 to 10 copies of viral RNA to be processed for detection quickly and with minimal use of equipment.

## 4. Application of Genome-Editing Proteins in Nucleic Acid Detection Assays

A more specific, sensitive, and rapid pathogen identification can be achieved by integrating the above-mentioned types of isothermal amplification with Cas-mediated detection methods. Over the last five years, plenty of new detection systems utilizing Cas proteins have been introduced. We attempt to systemize their parameters and possible application in Table 1. Cas proteins can help detect the majority of viral and bacterial pathogens in extremely low concentrations (see Table 1).

As can be seen from Table 1, the choice of a Cas-based detection system for a particular application depends on the type of nucleic acid and, consequently, on the detection object.

**Figure 5:**
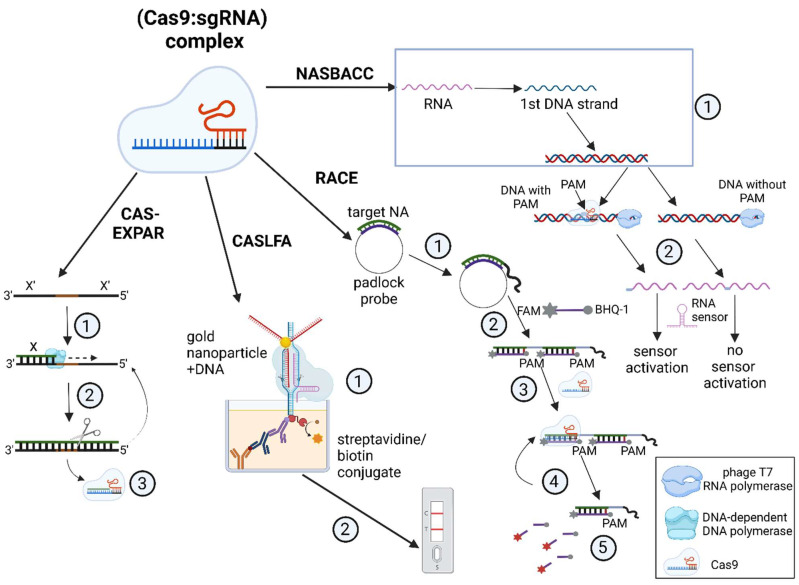
Nucleic acid detection methods using the Cas9 protein. **Cas–EXPAR**: (1) dsDNA synthesis combined with (2) its cleavage by a restriction enzyme; (3) Cas9-mediated interference. **CASLFA**: (1) formation of a complex conjugate due to the recognition of [gold nanoparticle: DNA] complex by Cas9 complex that is immobilized on the streptavidin/biotin conjugate; (2) lateral flow detection; **RACE**: (1) recognition of target nucleic acid by a padlock probe and (2) synthesis of the specific product by RCA; (3) FAM-labeled probe hybridization; (4) Cas9-mediated [probe: template] recognition and cleavage leading to (5) the emission of a fluorescent signal; **NASBACC**: (1) simplified outline of NASBA that produces dsDNA; (2) T7 RNA polymerase recognizes dsDNA sequences that, via PAM-specific cleavage, leads to the formation of two kinds of products: the truncated product mediates sensor activation, while the full-length one does not.

Due to the enzymatic cleavage activity of the Cas9 protein, Cas-based assays efficiently detect DNA/microRNA when used in combination with various isothermal amplification techniques and different methods of capturing specific signals (Figure 5).

For instance, in the CAS–EXPAR system, the Cas9 protein introduces a single-strand break, or nick, in a native DNA molecule and the nicked fragment is annealed to a DNA template. It is then extended by a DNA-dependent DNA polymerase in a strand-displacement reaction, and specific nicking endonucleases cleave the ssDNA fragment, leading to exponential accumulation of the cleaved product [7]. The detection limit for CAS–EXPAR reaches 0.82 aM, as determined by fluorescence analysis [7,57].

CASLFA simplifies the detection through immunochemical analysis in a lateral flow assay that is based on aurum nanoparticles (AuNP). Two molecular mechanisms can lead to the test line appearing on a test strip: either (1) the AuNP–DNA conjugate binds with an immobilized biotinylated non-target DNA strand (NTS), which is mediated by the interaction of the Cas9∙sgRNA complex with the target DNA strand (TS); or (2) the AuNP–DNA conjugate hybridizes with a stem-loop region sequence of the sgRNA when the complex binds anchored DNA [10]. The two ways can be mediated by both native Cas9 protein and its mutated variant dCas9 (deactivated Cas9) that lacks nuclease activity.

Alternatively, the RACE system is based on the RCA amplification method. It allows for the fluorescent detection of miRNAs that are only 19 to 23 nucleotides long, including oncogenic miRNAs. Detection is achieved by hybridizing a probe to a sequence amplified from the template miRNA, which is followed by the cleavage of this double-stranded region by CRISPR/Cas9 [11].

Finally, there are Cas9-based systems such as NASBACC that can assist in recognizing various SNPs in viral RNA [8]. The reaction mechanism depends on a specific toehold switch sensor that recognizes an RNA sequence produced via the NASBA reaction. In this case, the toehold switch can only sense full-sized transcripts that are amplified solely from DNA that is not cut by the Cas9–sgRNA complex owing to the absence of the PAM sequence. The downsides of this method are the long reaction time (3 h of NASBA followed by 30 min of Cas9-based detection) and the lack of a combined protocol for one-pot detection, which can result in contamination and false positives.

**Figure 6:**
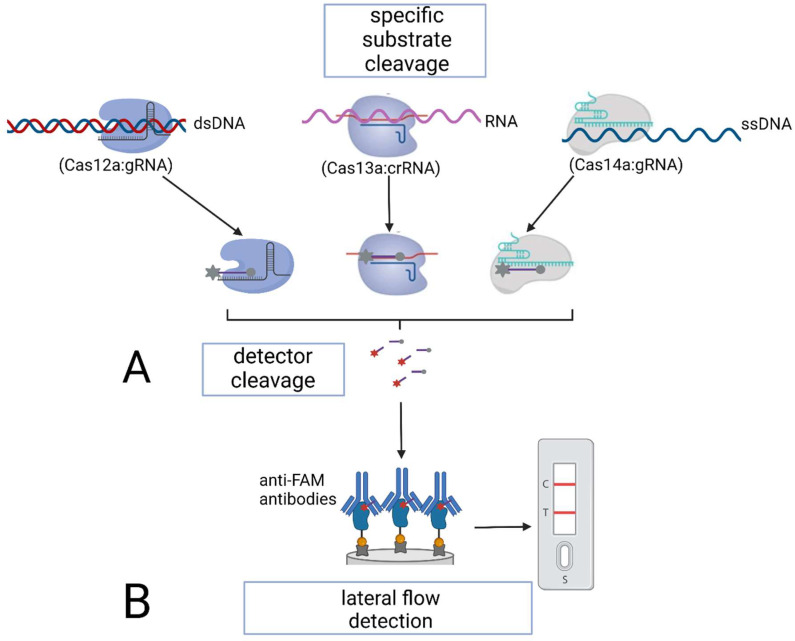
Specific nucleic acid detection using Cas12–Cas14 proteins: (**A**) via subsequent fluorescent detection; (**B**) via lateral flow detection.

Unlike Cas9-based detection systems, proteins Cas12, Cas13, and Cas14 possess non-specific cleaving activity that is triggered by the cleavage of a target sequence. Therefore, if genome-editing proteins of the Cas12–Cas14 family are used to detect nucleic acids after their isothermal amplification, the process consists of two stages: (1) specific cleavage of a target DNA or RNA; (2) cleavage of RNA/DNA probes followed by fluorescent detection (Figure 6).

Once the target sequence is cleaved, Cas12–Cas14 proteins develop non-specific, collateral cleavage activity. Thus, if the target RNA or DNA is present in the sample, Cas proteins cleave RNA/DNA probes—short (15 to 25 nucleotides) artificial RNA or DNA sequences carrying a fluorescent dye and a quencher. The dissociation of the dye and the quencher after cleavage triggers the fluorescence of the solution [13]. The quantitative fluorescent detection in this case is performed with a fluorescence reader within a relatively short time (less than 1 h).

Gootenberg et al. adapted the SHERLOCK platform for the detection of nucleic acids and developed SHERLOCK v.2 by introducing immunochromatographic analysis that relies on Cas13 and an auxiliary CRISPR-associated enzyme Csm6 [14]. SHERLOCK v2 utilizes an immunochromatographic lateral-flow detection method based on a specific activity of FAM-labeled RNA. These FAM reporters bind anti-FAM antibodies, which are conjugated with gold nanoparticles and anchored on a test strip, thus, preventing the conjugate from recognizing the bacterial protein A. When a FAM reporter is cleaved, the above-mentioned triple conjugates are formed and visualized as the sample (test) line in addition to the control line on a test strip. This modification of SHERLOCK provides even higher sensitivity due to a higher reaction rate, and the reagents are resistant to lyophilization, thus, making field-ready assays possible. Additionally, Joung et al. improved the method by combining isothermal amplification with Cas13-based detection in a one-pot reaction (SHERLOCK testing in one-pot) [51]. Positive samples can be detected within 15 to 45 min, while the specificity and sensitivity of the detection reach 98.5% and 93.1%, respectively.

Combinatorial arrayed reactions for multiplexed evaluation of nucleic acids (CARMEN) suggested by Ackermann et al. involve the Cas13a protein. This assay provides the conditions for multiplexed detection of nucleic acids in a single reaction with attomolar sensitivity. It has been applied to analyze a panel of more than 1000 samples containing 169 previously described viral RNAs. High throughput is achieved via an advanced signal detection technique: four commercially available fluorophores are combined to create highly specific color codes. Clinically, CARMEN allows for multiplexed detection of various viral pathogens in a biofluid sample. In particular, this method is proven to differentiate influenza virus serotypes (H1–H16, N1–N9) [13].

Chuan et al. optimized Cas12/Cas13-mediated detection by introducing a colorimetric approach that reliably detects traces of target nucleic acids (up to 10 copies) in clinical specimens within 3–5 min [52]. The colorimetric assay enables naked-eye visualization and involves complementary binding of linker DNA/RNA with specific probes conjugated with gold nanoparticles. Just as with SHERLOCK v.2, this method does not require any specialized equipment to analyze the results of an experiment.

Lopez-Valls et al. further optimized and modified this approach [53]. Their CRISPR/CAS-based colorimetric nucleic acid detection (CASCADE) method can detect picomolar concentrations of RNA without a preliminary isothermal amplification stage. If coupled with RPA or NASBA, its sensitivity covers the 3 fM and 40 aM ranges, respectively. Moreover, unlike the method described by Chuan et al. [52], CASCADE does not require centrifugation to achieve a qualitative change in the color of the reaction mix. This becomes possible due to extending the length of gold nanoparticle-conjugated oligonucleotides to 33 nucleotides, as well as increasing both the concentration of nanocomplexes and detection time.

An interesting approach to increasing the specificity of viral RNA detection in solutions is presented by Wang et al. [54]. They describe a CRISPR/Cas13-based CASCADE viral RNA assay for SARS-CoV-2 RNA detection. The assay is based on specific cleavage of SARS-CoV-2 RNA fragments and collateral cleavage of a hybrid nucleic acid pre-primer at its UU site to release an inactive phosphorylated primer. The primer is activated by dephosphorylation. Then, the primer binds the DNA sequence carrying T7-promoter and L-broccoli reporter gene and initiates chain extension to dsDNA mediated by the Klenow fragment without 5′–3′ exonuclease activity (exo-). This DNA serves as a template for transcription amplification by RNA polymerase, producing broccoli (RNA aptamers) that bind to the fluorescent dye DFHBI-1. The resulting complex emits a fluorescent signal. This method is highly sensitive (0.06 fM detection limit), but the presence of many enzymes in the assay mixture may trigger unwanted reactions.

Detection systems coupled with isothermal amplification are also available for the Cas14a protein that cleaves ssDNA sequences independent of PAM [55,58]. The Cas14–SDA method is a combination of SDA and specific Cas14a-mediated cleavage and it detects cholangiocarcinoma-associated miR-21 in concentrations as low as 680 fM [55]. The signal acquisition mechanism begins with hybridizing microRNA with a DNA template that contains the miRNA recognition site, a nicking (cleavage) site, and the Cas14a trans cleavage activation site. Bst polymerase recognizes the microRNA template complex and extends the sequences that are then cleaved by an endonuclease. Similarly to the Cas13a-mediated system, Cas14a is activated by amplified sequences, termed activators, when they are cleaved from long single-stranded nucleic acids (DNA in this case). Then, Cas14a cleaves the reporter FAM-labeled ssDNA oligonucleotides, and the fluorescent signal is emitted.

Thus, the systems based on isothermal amplification coupled with Cas-mediated interference reliably detect trace amounts of nucleic acids in biological specimens. These assays outperform alternative methods, such as PCR and real-time PCR, in detection speed, sensitivity, and ease of use. In addition to the concentration of the target DNA or RNA fragment, the most advanced detection systems also identify contaminants in the reaction, which reduces the time dedicated to optimizing reaction conditions.

## 5. Other Detection Methods Involving Cas Proteins

Although canonical systems that combine isothermal amplification and Cas-based detection are widely used in clinical and research studies, other approaches have been recently developed. Fozouni et al. exclude the isothermal amplification step and instead target multiple crRNAs at different sequences within a full-sized SARS-CoV-2 RNA [56]. Out of twelve crRNA molecules that target different genomic RNA regions of coronavirus, two crRNAs are chosen for the final assay since they generate the strongest fluorescent signal when they form a ribonucleoprotein complex with the Cas13a protein. This assay achieves 100 copies/μL sensitivity, and the detection time is 30 min, which allows the method to compete with isothermal-amplification-based assays in terms of usability and convenience. The specificity of this method enables differentiating SARS-CoV-2 RNA from RNA of other infectious agents, including MERS-CoV and influenza viruses A and B, which contribute the most to seasonal viral infections.

Finally, Iwasaki et al. demonstrate the feasibility of detecting low-molecular-weight compounds in solutions using a method termed SPRINT [16]. In its essence, SPRINT employs the activity of ‘riboswitches’—ligand-specific RNA sequences that recognize small compounds in solution (nucleotides, inorganic ions, etc.), thereby changing the secondary structure of RNA and making the target region available for Cas13a–crRNA-mediated cleavage. The cleavage triggers collateral RNase activity of Cas13a, as described above, and, hence, a specific fluorescent signal is generated by the cleaved reporter RNA oligonucleotides.

## 6. Conclusions

Cas proteins are increasingly gaining popularity as tools for the diagnostics of viral and bacterial infections due to their ability to recognize nucleic acids with high sensitivity and specificity. The collateral cleavage activity of Cas proteins is used to generate detection signals of various origins, such as fluorescent [13], immunochemical [14], or even colorimetric signal that allows for naked-eye visualization of the positive signal [53]. Furthermore, the broad adoption of isothermal amplification enables combining it with the detection techniques based on genomic-editing proteins. Isothermal amplification yields high concentrations of target nucleic acid fragments with no sophisticated specialized equipment required. It is also possible to carry out these combined reactions beyond the laboratory setting, which is also facilitated by the lyophilization stability of many components of these assays [13]. In addition, it is worth noting that methods that identify target nucleic acid sequences without their preliminary amplification are now actively being developed [56] to further simplify and accelerate the detection of pathogens in clinical and research settings.

## Figures and Tables

**Table 1:** Nucleic acid detection systems based on the activity of Cas proteins.

Detection System	CRISPR/Cas	Amplification	Target (DNA/RNA)	Detection Object	References
Cas type-II-based detection systems					
CAS-EXPAR	Cas9	PCR	dsDNA, miRNA	*L. monocytogenes*	[5]
NASBACC	Cas9	NASBA	dsDNA	Zika virus	[6]
CRISDA	Cas9	SDA	dsDNA	Human genomic DNA	[7]
CASLFA	Cas9 (dCas9)	RPA/PCR	dsDNA	*L. monocytogenes*, African swine fever virus	[8]
RACE	Cas9	RCA	miRNA	Vesicular miRNAs (miR-121, 122)	[9]
Cas type-V- and type-VI-based detection systems					
DETECTR	Cas12a	RPA	dsDNA	Human papillomavirus	[10]
HOLMES	Cas12a	PCR	dsDNA	Pseudorabies virus, Japanese encephalitis virus	[11]
HOLMES v.2	Cas12b	Asymmetric PCR, LAMP, RT–LAMP	dsDNA	Japanese encephalitis virus	[12]
SHERLOCK	Cas13a	RPA/RT–RPA	DNA/RNA	Zika virus, Dengue virus	[13]
SHERLOCK v.2	Cas13a, Cas13b, Cas12a	RPA/RT–RPA	DNA/RNA	Zika virus, Dengue virus	[14]
CARMEN	Cas13a	RPA/RT–RPA	DNA/RNA	169 human viruses	[15]
SPRINT	Cas13a	RPA/RT–RPA	DNA/RNA	Inorganic compounds, *B. subtilis*	[16]
SHINE	Cas13a	RPA/RT–RPA	DNA/RNA	SARS-CoV-2	[17]
DETECTR–Cas14	Cas14	LAMP	ssDNA	human *HERC2* gene	[43]
STOP	Cas13a	RPA/RT–RPA	DNA/RNA	SARS-CoV-2	[51]
Colorimetric detection	Cas12a/Cas13a	PCR, RPA	DNA/RNA	*A. tumefaciens,* African swine fever virus, *Listeria monocytogenes*, *Staphylococcus aureus*, *Neisseria encephalitis*, *Salmonella typhimurium*, *Enterobacter sakazakii*, *Pseudomonas aeruginosa*, *Vibrio parahemolyticus (*Cas12); miRNA, *L. monocytogenes*, *S. aureus*, *S. typhimurium, E. sakazakii*, *P. aeruginosa*, and *V. parahemolyticus* (Cas13)	[52]
CASCADE	Cas13a	RPA, NASBA	DNA/RNA	SARS-CoV-2	[53]
Cas13C	Cas13a	Combination of T7-transcription and synthesis of dsDNA by the Klenow fragment	RNA	SARS-CoV-2	[54]
Cas14SDA	Cas14a	SDA	miRNA	miR-21 in cholangiocarcinoma cells	[55]
Amplification-free detection	Cas13a	--------------	RNA	SARS-CoV-2, MERS-CoV, Influenza A and B viruses	[56]

## Data Availability

Data sharing not applicable.
